# Aqueous Extract of *Perilla frutescens* var. acuta Relaxes the Ciliary Smooth Muscle by Increasing NO/cGMP Content In Vitro and In Vivo

**DOI:** 10.3390/molecules23071777

**Published:** 2018-07-19

**Authors:** Jaeyong Kim, Huwon Kang, Hakjoon Choi, Ara Jo, Dooi-Ri Oh, Yujin Kim, Sojeong Im, Seul-Gi Lee, Kyeong-In Jeong, Geun-Chang Ryu, Chulyung Choi

**Affiliations:** 1 Jeonnam Institute of Natural Resources Research, Jangheung-gun, Jeollanamdo 59338, Korea; jykim761217@gmail.com (J.K.); whk0081@naver.com (H.K.); ohchj12@naver.com (H.C.); joara9153@naver.com (A.J.); pp0806@nate.com (D.-R.O.); ngel2327@naver.com (Y.K.); sojungbalm@naver.com (S.I.); thelsg@naver.com (S.-G.L.); 2 Department of Optometry and optic science Dong-Shin University, Naju 58245, Korea; yellow1011@naver.com (K.-I.J.); yoooptic@hanmail.net (G.-C.R.)

**Keywords:** *Perilla frutescens* var. acuta, eye fatigue, ciliary smooth muscle, NO, cGMP, luteolin-7-*O*-diglucuronide, apigenin-7-*O*-diglucuronide

## Abstract

The leaves of *Perilla frutescens* var. acuta (PFA) are commonly used as a traditional medicine in Korea, Japan, and China. We previously showed that PFA attenuates eye fatigue by improving visual accommodation through a clinical study. However, detailed mechanisms and chemical compounds have not been studied. In this study, we analyzed the active compounds in an aqueous extract of PFA involved in ciliary muscle relaxation in vitro and in vivo. NMR and MS analyses showed that the PFA extract contained mainly luteolin-7-*O*-diglucuronide and apigenin-7-*O*-diglucuronide. The composition after freeze-drying and spray-drying was similar. Freeze-dried PFA (50 µg/mL, 100 µg/mL, and 200 µg/mL) increased nitric oxide and cGMP levels in ciliary muscle cells isolated from the eyes of rats. [Ca^2+^]*i* decreased in a dose-dependent manner. Furthermore, Sprague-Dawley rats treated with freeze-dried PFA (200 mg/kg, orally) showed significantly increased cGMP levels compared with the control group and irradiated with white light. Our results suggest that PFA extract has the potential to reduce eye fatigue by relaxing ciliary muscles.

## 1. Introduction

The leaves of *Perilla frutescens* var. acuta (PFA) has been used herbal medicines in the treatment of depression, anxiety, allergies, bacterial and fungal infections, the common cold, shivering fits, fever, chest pains, and coughs in Asian countries [1,2,3,4]. The leaves of this plant are widely used as food material such as in sushi, soups, salads, spices, and food colorants [4]. Previous studies have stated that PFA exerts antioxidant [5], anti-glycosuria [4], anti-inflammatory [6], anti-allergic [7], antimicrobial [8], anti-dementia [9], anti-depressive [10], and anti-tumor effects [6]. The known chemical compounds of this plant include phenolic compounds, flavonoids, anthocyanin, essential oil, and phenylpropanoids [4,11]. The polyphenols contained in the plant extracts alleviate eye fatigue in humans [12]. However, the effects of PFA extracts and its components on eye fatigue have not been reported.

Recently, ocular fatigue associates with the use of visual display terminals (VDT) including computers and smartphones [13,14,15]. Overuse of such devices causes exhaustion of the ciliary muscle, which controls the thickness of the lens and decreases the accommodative ability of the eye [16].

Natural extracts of plants including those of biberry, *Haematococcus*, pluvialis, and black soybean hull have been reported to be effective in improving eye fatigue by controlling the ciliary muscle function in the human eye [17,18,19]. Moreover, natural compounds astaxanthin and anthocyanin improve visual accommodation by relaxing the ciliary muscles [17,20].

Ciliary smooth muscle relaxation is known to be regulated by both cAMP-dependent mechanisms such as prostaglandin receptor-mediated relaxation and cAMP-independent mechanisms such as nitric oxide-mediated relaxation [21]. Therefore, an increase in cAMP and cGMP contents can induce the relaxation of smooth muscles in the eye [21].

Nitric oxide (NO) is synthesized from L-arginine by NO synthase and is known to relax vascular smooth muscle through the elevation of cGMP that, in turn, mediates a relaxation response [22,23]. NO synthase has been detected in ocular structures such as the ciliary muscle [23]. In the eye, NO is an important factor that mediates visual accommodation and relaxes the contracted ciliary muscle [24]. Ciliary contraction could be induced by a cholinergic agent such as carbachol [25]. Additionally, previous studies have demonstrated that the [Ca^2+^]*i* concentration in cultured monkey smooth muscle cells is mainly a factor affecting the contraction of ciliary smooth muscle [26]. Carbachol increases the Ca^2+^ concentration in a dose-dependent and time-dependent manner in human ciliary muscle cells [27].

We previously showed that PFA improves an accommodative ability due to its relaxing effect on the ciliary muscle through clinical studies [28,29]. In this study, we evaluated the relaxing effects of PFA on cGMP and cAMP contents and Ca^2+^ mobilization in rat ciliary smooth muscle cells (rCSMCs) to confirm the mechanism to support our clinical observations. Lastly, the effect of PFA on cGMP, which is a relaxation factor, was studied using in vivo models. In addition, we aimed to analyze the phytochemical compounds of PFA.

## 2. Results and Discussion 

### 2.1. Preparation and Characterization of PFA

**Figure 1:**
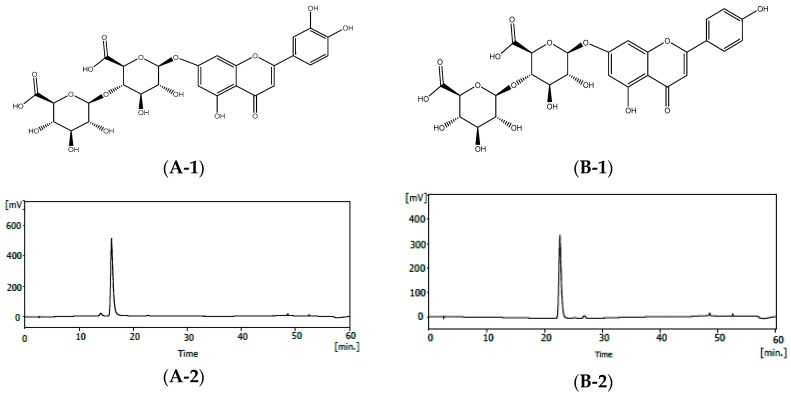

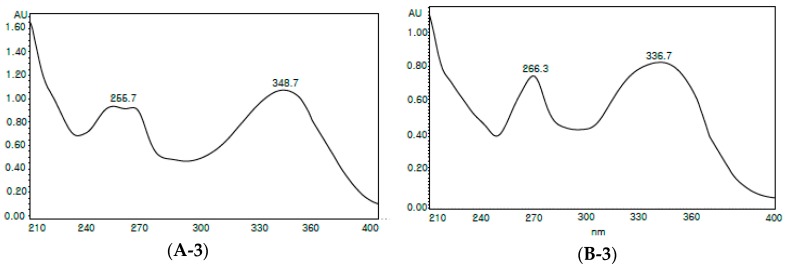
The structure and purity of luteolin-7-*O*-diglucuronide and apigenin-7-*O*-diglucuronide **f**rom *Perilla frutescens* var. acuta (PFA). (**A-1**) The structure of luteolin-7-*O*-diglucuronide. (**A-2**) HPLC profile of luteolin-7-*O*-diglucuronide. (**A-3**) The UV spectrum of luteolin-7-*O*-diglucuronide. (**B-1**) The structure of apigenin-7-*O*-diglucuronide. (**B-2**) HPLC profile of apigenin-7-*O*-diglucuronide. (**B-3**) The UV spectrum of apigenin-7-*O*-diglucuronide.

When comparing the above data with literature values [30,31], compound **1** and compound **2** were identified as luteolin-7-*O*-diglucuronide and apigenin-7-*O*-diglucuronide [31,32] (Figure 1). Aqueous extracts of PFA contain polyphenolic compounds such as gallic acid, caffeic acid, rosmarinic acid, flavonoids, scutellarein 7-*O*-diglucuronide, and anthocyanins [33]. Mainly, studies on their antioxidant effects have been carried out [4,33]. Rosmarinic acid in PFA has also been studied [34,35].

### 2.2. HPLC Analysis of Freeze-Dried and Spray-Dried Samples of PFA

**Figure 2:**
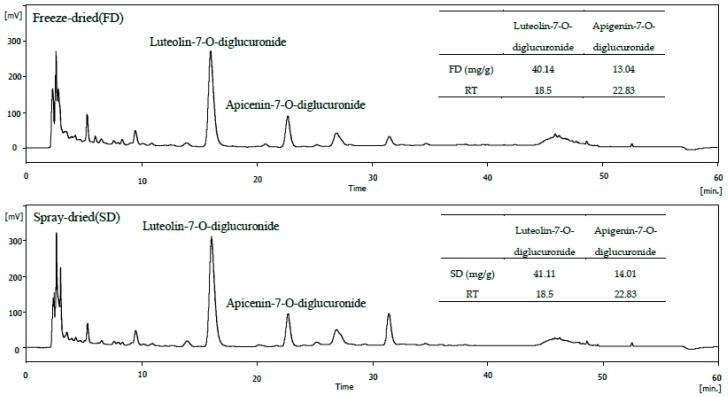
High-performance liquid chromatography of freeze-dried (FD) and spray-dried (SD) sample of PFA.

Using HPLC, we verified the difference in composition according to the two methods (freeze-drying versus spray-drying). As shown in Figure 2, the HPLC patterns of the freeze-dried and spray-dried formulations were similar. The polyphenol compounds were identified as luteolin-7-*O*-diglucuronide and apigenin-7-*O*-diglucuronide. Approximately, 40.14 and 41.11 mg/g luteolin-7-*O*-diglucuronide and 13.04 and 14.01 mg/g apigenin-7-*O*-diglucuronide were present in freeze-dried and spray-dried samples, respectively.

In the food industry, the drying step has a significant influence on the cost-effectiveness, functionality, and bioactive potential of the final product [30]. Correia et al. [30] reported that spray-dried and freeze-dried blueberry extracts showed similar phytochemical composition. Therefore, we used the freeze-dried sample that was used previously in our clinical trials because there was no change in the main composition depending on the drying method in this experiment. Furthermore, we confirm whether spray-drying can replace freeze-drying.

### 2.3. Effect of the Freeze-Dried PFA on NO Production in rCSMCs

**Figure 3:**
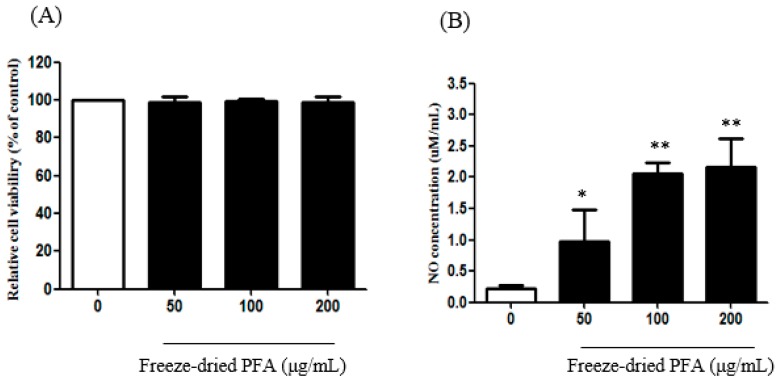
Effect of freeze-dried *Perilla frutescens* var. acuta (PFA) on (**A**) cell viability and (**B**) nitric oxide (NO) in rat ciliary smooth muscle cells. rCSMCs were treated with 50 μg/mL to 200 μg/mL of the freeze-dried PFA for 24 h. Cytotoxicity was estimated by the MTT assay and NO was quantified using the Griess agent. The data are represented as mean ± SD. * *p* < 0.05 and ** *p* < 0.01.

rCSMCs were treated with freeze-dried PFA at concentrations ranging from 50 µg/mL to 200 µg/mL for 24 h. There were no significant alterations in cell viability following freeze-dried PFA treatment at these concentrations (Figure 3A). Therefore, the cells were treated with 50 µg/mL, 100 µg/mL, and 200 µg/mL of the freeze-dried PFA extract to determine NO production. The freeze-dried PFA extract increased NO production compared to that in untreated cells in a dose-dependent manner (Figure 3B).

NO is a major factor involved in functions such as immune function regulation, ciliary muscle relaxation, and vasodilation [22,36]. Gabelt et al. [25] reported that NO mediates the modulation of ciliary muscle tension. NO induced ciliary smooth muscle relaxation via an increase in the cyclic GMP level [37]. Therefore, our results showed that freeze-dried PFA is an important inhibitory factor in the regulation of ciliary muscle tension.

### 2.4. Effect of the Freeze-Dried PFA on the Production of cAMP and cGMP in rCSMCs

**Figure 4:**
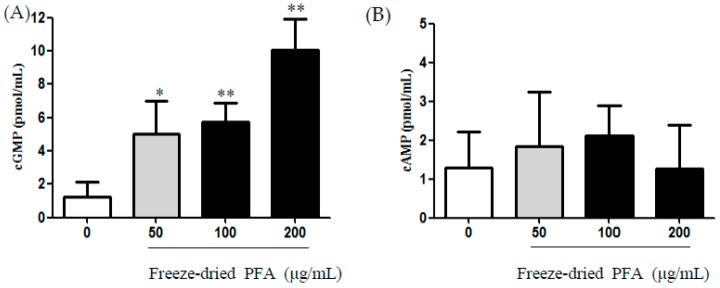
Effect of freeze-dried *Perilla frutescens* var. acuta (PFA) on cGMP and cAMP contents in rat ciliary smooth muscle cells. (**A**) Intracellular concentration of cGMP after 15 min of freeze-dried PFA treatment at 50 µg/mL, 100 µg/mL, and 200 µg/mL. (**B**) Intracellular concentration of cAMP after 15 min of freeze-dried PFA treatment at 50 µg/mL, 100 µg/mL, and 200 µg/mL. The data are represented as mean ± SD. * *p* < 0.05 and ** *p* < 0.01.

Treatment of rCSMCs with freeze-dried PFA at a dose of 50 µg/mL, 100 µg/mL, and 200 µg/mL significantly increases the contents of intracellular cGMP with stimulation observed 15 min after the addition of freeze-dried PFA in a concentration-dependent manner (Figure 4A). However, freeze-dried PFA did not affect cAMP content at 50 µg/mL, 100 µg/mL, and 200 µg/mL (Figure 4B). These results indicated that freeze-dried PFA directly increases cGMP levels. Kamikawatoko et al. [23] reported that smooth muscle relaxation occurs through cGMP activation. Matsumoto et al. [38] reported that delphinidin-3-rutinoside induces relaxation by elevating cGMP content in the bovine ciliary smooth muscle. In our previous studies, we examined the effects of PFA on PDE5A and PDE3A inhibitory activity using smooth muscle cells [39]. PFA significantly inhibited PDE5A activity in a dose-dependent manner, but no inhibitory effect was observed on PDE3A activity. Therefore, we did not confirm this activity in the present study. It is necessary to identify factors in PFA extracts that lead to an eNOS-dependent increase in cGMP. However, we focused on the main factors such as NO, cGMP, cAMP, and [Ca^2+^]*i* of smooth muscle cells because ciliary smooth muscle cells included smooth muscle. Based on our experimental results, it is conceivable that the PFA extract induces NOS in endothelial cells. We will carry out experiments to evaluate an eNOS-dependent increase in cGMP by PFA. Therefore, freeze-dried PFA may relax the ciliary muscle through the NO/cGMP pathway.

### 2.5. Effect of the Freeze-Dried PFA Extract on [Ca^2+^]i in rCSMCs

**Figure 5:**
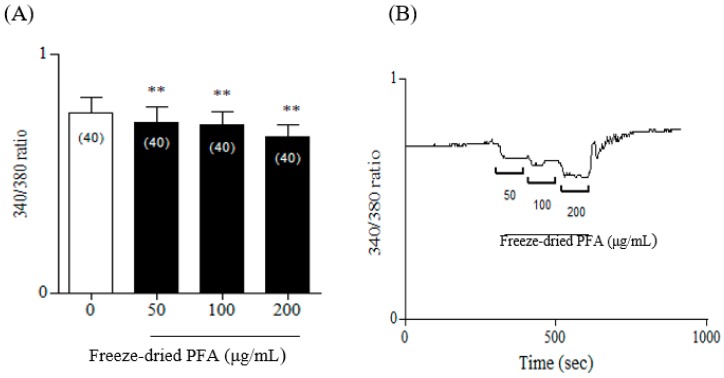
Effect of freeze-dried *Perilla frutescens* var. acuta (PFA) on basal [Ca^2+^]*i* in rat ciliary smooth muscle cells. (**A**) Representative tracing of the [Ca^2+^]*i* response evoked by various concentrations of freeze-dried PFA (50 µg/mL, 100 µg/mL, and 200 µg/mL). (**B**) The panel shows the summarized data of freeze-dried PFA-induced changes in [Ca^2+^]*i* in rat ciliary smooth muscle cells. The experimental numbers are given in parentheses. The data are represented as mean ± SD. * *p* < 0.05 and ** *p* < 0.01.

Freeze-dried PFA (0 µg/mL, 50 µg/mL, 100 µg/mL, and 200 µg/mL) were measured on rCSMCs to confirm whether Freeze-dried PFA inhibited [Ca^2+^]*i* contents using Fura-2 ratiometric Ca^2+^ imaging. Freeze-dried PFA caused a rapid decrease in [Ca^2+^]*i* in these cells compared with the control (Figure 5). Therefore, freeze-dried PFA substantially decreased [Ca^2+^]*i* concentrations in a dose-dependent manner.

Contractility of the ciliary muscle is regulated by calcium ions [26]. Matsumoto et al. [27] reported that muscarinic agonists such as carbachol increase [Ca^2+^]*i* in human ciliary smooth muscle cells. Therefore, freeze-dried PFA may play a major role in regulating calcium ions in ciliary muscle. In further studies, the mechanism underlying the inhibitory effects of freeze-dried PFA in ciliary muscles should be elucidated.

### 2.6. Effect of the Freeze-Dried PFA Extract on the Production of cGMP In Vivo

**Figure 6:**
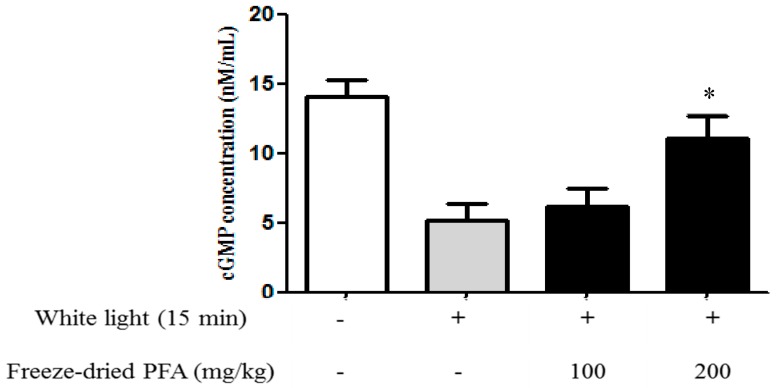
Effect of freeze-dried *Perilla frutescens* var. acuta (PFA) on cGMP contents in rat whole eye. Freeze-dried PFA was administered at 100 mg/kg and 200 mg/kg doses for three days before irradiation with white light for 15 min. Intracellular cGMP levels were measured by ELISA. The data are represented as mean ± SD (*n* = 5). * *p* < 0.05 compared with the control.

To verify if in vitro results could be replicated in vivo, we studied the effect of freeze-dried PFA (100 mg/kg and 200 mg/kg) on cGMP levels in white light-induced eye fatigue animal experiments. Freeze-dried PFA (200 mg/kg) significantly increased intracellular cGMP levels in rats irradiated with white light compared with the control (Figure 6).

Hiramoto et al. [40] reported that white light irradiation of the eye causes eyestrain and fatigue. Notably, our study is the first to report on animal models of eye fatigue. In our previous clinical test, the PFA intake group significantly improved visual fatigue along with an accommodative ability [28,29]. In further studies, animal models of visual fatigue that are relevant to a clinical study should be investigated.

## 3. Materials and Methods

### 3.1. Materials

Dulbecco’s modified Eagle’s medium/Ham’s F12 (DMEM/F12) medium and fetal bovine serum were purchased from Invitrogen-Gibco (Grand Island, NY, USA). The Papain dissociation system was purchased from Worthington Biochemical Co. (Lakewood, NJ, USA). The cGMP ELISA kit was purchased from Cell Biolabs Inc. (San Diego, CA, USA) and cAMP ELISA kit was purchased from R&D systems (Minneapolis, MN, USA). Fura-2/AM and Pluronic F-127 were obtained from Molecular Probes (Eugene, OR, USA). Other chemicals were purchased from Sigma-Aldrich (St. Louis, MO, USA).

### 3.2. Extraction and Drying Process

Dried leaves of PFA were purchased from cultivated fields in Jecheon, Chungbuk, Korea. The plant was identified by a researcher of botany from the Jeollanamdo Institute for Natural Resources Research, Jangheung, Korea [41]. PFA leaves were extracted in distilled water. The water extract was concentrated under a reduced pressure (Buchi, Flawil, Switzerland). The solution was either freeze-dried or spray-dried, respectively.

### 3.3. Indentification of Compounds

To isolate the major chemical compounds, the PFA extract (2 L) was subjected to separation on a Diaion HP-20 column (Mitsubishi Chemical Co., Tokyo, Japan) and eluted with H_2_O/MeOH (30:70, 50:50, 70:30, 0:100, each 2 L). Lastly, it was washed by acetone (2 L) to give five fractions. Among the five fractions, fraction 1, which contained active or major compounds in abundance, was purified by preparative Waters HPLC with a YMC-triart C18 column (10 mm × 200 mm, 5 μm particle size) and separated using a gredient elution. The mobile phase was composed of MeOH (A) and 0.5% formic acid in water (B). It was started with 25% to 100% solvent A for 50 min at a flow rate of 2 mL/min. Compound 1 (150 mg, purity 96.7%) and compound 2 (50 mg, purity 96.4%) were detected at 254 nm, respectively. Compounds **1** and **2** were identified to be luteolin-7-*O*-diglucuronide and apigenin-7-*O*-diglucuronide, respectively, by NMR and MS analyses. An Avance-500 spectrometer (Bruker Corporation, Billerica, MA, USA) was used to record Nuclear Magnetic Resonance (NMR) spectra (500 MHz) with deuterated dimethyl sulfoxide (DMSO) as NMR solvent. Mass spectra were obtained on a Shimadzu UFLC(XR)-20A (Dong-il SHIMADZU Corp. Kyoto, Japan). 

*Compound***1**, buff powder, ESI-MS: 639 (M + H)^+^. ^1^H-NMR(500 MHz, pyridine-*d*5) δ: 4.25~4.74(1H, H-1 GluA2), 4.38~4.76 (1H, d, *J* = 7.2 Hz, H-1 GluA1), 4.92 (1H, d, *J* = 9.5 Hz, H-5′′), 5.57 (1H, d, *J* = 8 Hz, H-1′′′), 6.05 (1H, d, *J* = 7 Hz, H-1′′), 6.82 (1H, s, H-3), 7.13 (1H, d, *J* = 2 Hz, H-6), 7.17 (1H, d, *J* = 2 Hz, H-8), 7.21 (1H, d, *J* = 8.5 Hz, H-5′), 7.43 (1H, dd, *J* = 2.0, 8.5 Hz, H-6′), 7.85 (1H, d, *J* = 2 Hz, H-2′). ^13^C-NMR (125 MHz, pyridine-*d*5) δ: 72.43 (C-4′′), 73.16 (C-4′′′), 76.02 (C-2′′′), 76.80 (C-5′′), 77.40 (C-3′′), 77.62 (C-3′′′), 78.06 (C-5′′′), 83.89 (C-2′′), 95.61 (C-8), 100.06 (C-1′′), 100.64 (C-6), 103.77 (C-3), 106.57 (C-1′′′), 106.75 (C-10), 114.49 (C-2′), 116.57 (C-5′), 119.47 (C-6′), 122.46 (C-1′), 147.47 (C-3′), 151.61 (C-4′), 157.55 (C-5), 162.46 (C-9), 163.47 (C-7), 165.00 (C-2), 171.79 (C-6′′′), 172.37 (C-6′′′), 182.61 (C-4).

*Compound***2**, buff powder, ESI-MS: 623 (M + H)^+^. ^1^H-NMR(500 MHz, pyridine-*d*5) δ: 4.25~4.65(1H, H-1 GluA2), 4.38~4.77 (1H, d, *J* = 7.2 Hz, H-1 GluA1), 4.95 (1H, d, *J* = 7 Hz, H-5′′), 5.58 (1H, d, *J* = 8 Hz, H-1′′′), 6.08 (1H, d, *J* = 7 Hz, H-1′′), 6.81 (1H, s, H-3), 7.13 (1H, d, *J* = 2 Hz, H-6), 7.16 (1H, d, *J* = 2 Hz, H-8), 7.20 (1H, d, *J* = 8.5 Hz, H-5′), 7.29 (1H, dd, *J* = 2.0, 8.5 Hz, H-6′), 7.80 (1H, d, *J* = 8.5 Hz, H-2′). ^13^C-NMR (125 MHz, pyridine-*d*5) δ: 72.46 (C-4′′), 73.16 (C-4′′′), 76.02 (C-2′′′), 76.83 (C-3′′), 77.43 (C-5′′), 77.63 (C-3′′′), 78.06 (C-5′′′), 83.95 (C-2′′), 95.60 (C-8), 100.06 (C-1′′), 100.78 (C-6), 103.69 (C-3), 106.57 (C-10), 106.79 (C-1′′′), 116.57 (C-3′), 116.57 (C-5′), 121.82 (C-1′), 128.74 (C-2′), 128.74 (C-6′), 157.56 (C-5), 162.46 (C-9), 162.52 (C-4′), 163.58 (C-7), 164.63 (C-2), 171.78 (C-6′′′), 172.36 (C-6′′′), 182.64 (C-4). 

### 3.4. HPLC Analysis

High performance liquid chromatography (HPLC) analysis of the powders obtained in the freeze-dried and spray-dried of PFA. The analysis was performed using the Waters series HPLC system (Waters corporation 34 Maple street Milford, MA, USA) comprised of a binary pump (1525), a photodiode array detector (2998), and an auto injector (2707). The column was a Triart-C18 (250 mm × 4.6 mm, 5 μm, YMC, Kyoto, Japan) and the detection wavelength were set at 254 nm for water extract, luteolin-7-*O*-diglucuronide, and apigenin-7-*O*-diglucuronide. The column thermostat was maintained at 35 °C. Mobile phase A was methanol and mobile phase B was water (containing 0.5% formic acid) with the elution profile as follows: 0–10 min, 30% A, 10–30 min, 30–40% A, 30–40 min, 40–50% A, 40–45 min, 50–100% A, 45–53min, 100% A, 53–56 min 100–30% A, 56–60 min, 30%. The flow rate was 1 mL/min and the injection volume was 20 uL [39].

### 3.5. Preparation of Tissue and Cell Culture

rCSMCs were obtained from 3–4-week-old male Sprague-Dawley rats. The ciliary body cell culture was performed using a modified papain dissociation system (Worthington Biochemical Co., Lakewood, NJ, USA). The eyes were removed and cut in half. The corneal portion of the cut eyes was placed in a vial containing a papain solution and is incubated at 37 °C with constant agitation for 90 min. The cloudy cell suspension was transferred to a sterile tube and centrifuged at 300× *g* for 5 min at room temperature. After discarding the supernatant, the pelleted cells were immediately resuspended in DMEM/F-12 (Invitrogen-Gibco, Grand Island, NY, USA). The dissociated ciliary muscle cells were cultured in DMEM/F-12 medium with 10% fetal bovine serum (FBS) for 5 days to 2 weeks [42].

### 3.6. Cell Viability

rCSMCs were plated at 1 × 10^4^ cells/well in 96-well plates and treated with freeze-dried PFA at 50 µg/mL, 100 µg/mL, and 200 µg/mL for 24 h. Then, 100 μL of WST-1 solution was added to each well and the plates were incubated for 2 h at 37 °C, according to the protocol of the WST-1 assay kit. The cell viability measured for the absorbance at 450 nm on a microplate reader (Bio-Rad, Hercules, CA, USA).

### 3.7. Determination of NO Production

rCSMCs (5 × 10^5^ cells/mL) plated in 96-well plates were pre-incubated for three days. Then, the freeze-dried PFA extract (50 µg/mL, 100 µg/mL, and 200 µg/mL) was added to the cells and the cells were incubated at 37 °C for 24 h. The level of NO production was determined by assaying the culture supernatants for nitrite, which is the stable product of the reaction between NO and the Griess reagent. The nitrite concentration was obtained by measuring the absorbance of standard solutions of nitrate sodium at 540 nm on a microplate reader [43].

### 3.8. Measurement of cGMP and cAMP Production

rCSMCs were plated at 1~2 × 10^5^ cells/well for 5 days in the absence of freeze-dried PFA. After 5 days, the cells were pretreated PBS containing 1 mM 3-isobutyl-1-methylxanthine, a phosphodiesterase inhibitor for 10 min, and treated with freeze-dried PFA (50 μg/mL, 100 μg/mL, and 200 μg/mL) for 15 min. The medium was aspirated and the intracellular cGMP and cAMP contents of each sample were determined by ELISA [39].

### 3.9. Measurement of [Ca^2+^]i

Fura-2/AM was used as the fluorescent marker of [Ca^2+^]*i*. rCSMCs were incubated for 60 min at room temperature with HEPES buffer (155 mM NaCl, 3 mM KCl, 2 mM CaCl_2_, 10 mM HEPES, 10 mM glucose, and 1 µM glycine; pH 7.4) containing Fura-2/AM (5 µM) and Pluronic F-127 (0.001%). The rCSMCs were stabilized in HEPES-buffer for 5 min, which was followed by freeze-dried PFA treatment (50 µg/mL, 100 µg/mL, and 200 µg/mL) for 100 s to confirm changes in [Ca^2+^]*i* contents and then the cells were illuminated using Lambda XL at excitation wavelengths of 340 nm and 380 nm. Data were acquired every 2 s. All imaging data were collected and analyzed using MetaMorph software [27].

### 3.10. Measurement of cGMP Production In Vivo

Male Sprague-Dawley rats (5–6 weeks, 180–200 g) were purchased from Samtako (Osan-si, Gyeonggi-do, Korea). The animal room environment was maintained at a temperature of 22 ± 3 °C, relative humidity of 50 ± 20%, ventilation of 10–15 air changes per hour, and a 12-h light/dark cycle. Animals were provided food and water ad libitum. The rats were then assigned randomly to four groups. The normal group received distilled water for three days without white light and the control group received distilled water irradiated with white light (100 W) for 15 min. Freeze-dried PFA was administered orally at 100 mg/kg and 200 mg/kg for three days. Freeze-dried PFA was orally administered on day 3 and then the rats were irradiated with white light (100 W) for 15 min. After sacrifice, their eyes were immediately enucleated and washed in PBS. After homogenization and centrifugation, the supernatant was analyzed using a cGMP ELISA kit.

Our animal study was approved by the Institutional Animal Care and Use Committee (IACUC) at the Jeollanamdo Institute for Natural Resources Research (approval No. JINR1801). All animal experiments were conducted in accordance with the IACUC guidelines.

### 3.11. Statistical Analysis

The results are expressed as a mean ± SD. A comparison between groups was performed using one-way ANOVA. Differences between individual treatment groups were compared using the Dunnett’s test. Statistical significance was set at *p* < 0.05 and *p* < 0.01. The statistical analyses were performed using the GraphPad Prism software version 5.0.

## 4. Conclusions

Our results show that freeze-dried PFA significantly increases the levels of NO, cGMP, and [Ca^2+^]*i* in rCSMCs. Additionally, in vivo results supported the role of freeze-dried PFA in reducing eye fatigue by increasing cGMP. The active compounds were identified as luteolin-7-*O*-diglucuronide and apigenin-7-*O*-diglucuronide by NMR. The HPLC patterns of freeze-dried and spray-dried samples of PFA were similar. In the future, the physiological activity of luteolin-7-*O*-diglucuronide and apigenin-7-*O*-diglucuronide may be evaluated.
